# Extirpation of the Entire Clavicle

**Published:** 1858-01

**Authors:** 


*Extirpation of the entire clavicle.*—Dr. C. R. S. Custis of Chicago reports (Am. Jour. Med. Sci.) a case of successful removal of the entire clavicle, for an osteo-sarcomatous tumor, involving its middle third and external portion of sternal extremity. The patient had previously had syphilis.—*Ib.*

